# Tomato as a Source of Carotenoids and Polyphenols Targeted to Cancer Prevention

**DOI:** 10.3390/cancers8060058

**Published:** 2016-06-20

**Authors:** Raúl Martí, Salvador Roselló, Jaime Cebolla-Cornejo

**Affiliations:** 1 Unidad Mixta de Investigación Mejora de la Calidad Agroalimentaria UJI-UPV, Department de Ciències Agràries i del Medi Natural, Universitat Jaume I, Avda. Sos Baynat s/n, 12071 Castelló de la Plana, Spain; martir@uji.es (R.M.); rosello@uji.es (S.R.); 2 Unidad Mixta de Investigación Mejora de la Calidad Agroalimentaria UJI-UPV, COMAV, Universitat Politècnica de València, Cno., De Vera s/n, 46022 València, Spain

**Keywords:** breeding, functional food, lycopene, β-carotene, flavonoid, anthocyanin

## Abstract

A diet rich in vegetables has been associated with a reduced risk of many diseases related to aging and modern lifestyle. Over the past several decades, many researches have pointed out the direct relation between the intake of bioactive compounds present in tomato and a reduced risk of suffering different types of cancer. These bioactive constituents comprise phytochemicals such as carotenoids and polyphenols. The direct intake of these chemoprotective molecules seems to show higher efficiencies when they are ingested in its natural biological matrix than when they are ingested isolated or in dietary supplements. Consequently, there is a growing trend for improvement of the contents of these bioactive compounds in foods. The control of growing environment and processing conditions can ensure the maximum potential accumulation or moderate the loss of bioactive compounds, but the best results are obtained developing new varieties via plant breeding. The modification of single steps of metabolic pathways or their regulation via conventional breeding or genetic engineering has offered excellent results in crops such as tomato. In this review, we analyse the potential of tomato as source of the bioactive constituents with cancer-preventive properties and the result of modern breeding programs as a strategy to increase the levels of these compounds in the diet.

## 1. Introduction

Traditionally, the concept of quality in vegetables has been related with their external appearance. The main requirements of food industries and markets were focused on the demand of new varieties with higher uniformity, brighter colours and enhanced shelf life as essential characteristics. Following these market requirements, traditional breeding programs focused their attention in these external features together with higher yields and disease resistance, neglecting other important aspects such as flavour or functional characteristics. Over the past decades, flavour was reconsidered in breeding programs due to the continuous complaints of consumers about the loss of traditional organoleptic characteristics of vegetables [1]. More recently, following this lead, health benefits of food have also started to be valued [2]. Nowadays, consumers are aware of the functional characteristics of agricultural food products, and more consumers choose foods considering their healthy characteristics. Although it is not clear whether marketing or for health functionality is spurring this interest, or vice versa, there is an increasing attention in the development of new phytochemical-rich vegetable varieties via breeding programs [3].

It is a well-known fact that fruits and vegetables are an important source for the daily intake of healthy constituents to the diet like minerals (calcium, phosphorous, magnesium and other minor minerals), water-soluble vitamins (B and C), fat-soluble vitamins (A, E and K) and a wide variety of phytochemicals [4]. Among phytochemicals, we can find bioactive molecules capable to protect against diseases acting as free radical scavengers or antimicrobial agents. The most renowned phytochemicals from vegetables are polyphenols, carotenoids, organo-sulfur and seleno-compounds. Their presence in certain plants would, in part, justify the epidemiologic evidence of a protective role of diets rich in fruits, vegetables, legumes and whole grains [5].

The traditional Mediterranean diet would be an example of how a diet characterized by a high consumption of foods of plant origin, relatively low consumption of red meat and high consumption of olive oil results in reduced occurrence of cancer [6], coronary heart disease and other diet-related chronic diseases [7]. Among the components of the Mediterranean diet, fruits and vegetables outstand in their preventive role. In Western countries, such as in the United States, a goal of at least 9 to 13 servings a day of fruits and vegetables is promoted, though the real consumption does not reach half that Figure [8]. This goal has proved to be quite difficult to reach, as studies developed in the late 90s and early 2000s pointed out [9]. Increasing awareness of the need for consumption of these foods and a complete educational model starting at nursery schools would be required.

Although the bioactive constituents of fruits and vegetables may be delivered via dietary supplements, scientific evidences seem to point out that their direct intake in their natural matrix has more health benefits than if those chemoprotective molecules are ingested isolated or in dietary supplements [10]. In that line, some authors pointed out that whole tomato consumption is more effective than lycopene isolated for the prevention and the development of progression of prostate cancer in rats [11]. According to this, more researches suggested that the synergetic effects of these molecules with other phytochemicals presents in vegetables may enhance their health benefits [12,13]. Thus, a diet rich in vegetables is the best way to obtain a wide variety of phytochemicals [8]. It should be considered, though that in the case of liposoluble compounds the diet should be complemented with oils. It would be the case of carotenoids. Most fruits and vegetables rich in carotenes are low in lipids. Therefore, in order to promote their absorption, it would be necessary to promote the joint intake of lipids. For example, the addition of avocado to salads has proved to improve the absorption of tomato carotenoids [14] or in the case of cooked tomato, the use of olive oil in the cooking process would also greatly increase the absorption of lycopene [15].

We should be aware of the difficulty of clearly establishing the protective role of a certain compound in a complex diet over the development of chronic diseases, which are affected by many other risk factors. In this context, some studies have questioned the relation between the ingestion of vitamin C or carotenoids and a reduction in the risk of suffering cancer [16,17] while other studies continue to establish this relation [18,19]. Apart from the difficulty of establishing the role of a single component of a complex diet certain considerations such as the measurement errors in the dietary intake of fruit and vegetables may also attenuate the relationships observed in epidemiological studies [20].

In this complex context, there is an increasing interest in the development of new vegetable varieties with increased levels of bioactive components [3], in order to promote their protective role in well balanced diets such as the Mediterranean. Although, it is necessary to continue on research of the effects of phytochemicals on health to understand the mechanism of action and the associations of these molecules in cancer-risk prevention, the development of long-term breeding programs should be started to promote the benefits of vegetable consumption.

The high level of consumption and the high economic value of tomato production has spurred the scientific and breeding efforts in this species. Although tomato does not outstand for its high concentration in bioactive compounds, the high levels of tomato intake both fresh and processed, position tomato as one of the main sources of chemoprotective compounds to diet [21], including vitamin C. Although tomato cultivars with increased vitamin C contents have been developed, vitamin C content is rather unstable, as it is highly dependent on the environment (especially via genotype x environment interactions) and it is quickly used as antioxidant under stress conditions. Thus, its management in breeding programs can be quite complicated [22]. Apart from vitamin C, the main bioactive compounds present in tomato (*Solanum lycopersicum* L.) are carotenoids and polyphenols. Here we review the chemoprotective characteristics of these last tomato bioactive compounds, their biosynthesis and the achievements in breeding programs targeted to increase their contents.

## 2. Accumulation of Bioactive Compounds in Tomato

In tomato, carotenoids are synthesized in the leaves, flowers and fruits. In the leaf tissues, carotenoids act as photoprotectors [23], being lutein the main carotenoid present meanwhile the presence of the xanthopylls violaxanthin and neoxanthin confer the characteristic yellow colouration to flowers [24]. In ripe tomato fruits, lycopene is the main carotenoid that can be found and it causes its red colouration (Table 1).

The contents of carotenoids, as well as other chemoprotective substances are highly conditioned by the genotype and environmental conditions (reviewed by Tiwari and Cummings [28]). Considering this variability, lycopene concentrations from standard tomato cultivars range from 7.8 to 18.1 mg 100 g^−1^ fresh weight (fw) (Table 1). Other colourless intermediates from the carotenoid biosynthetic pathway may be found in tomatoes. This is the case of phytoene and phytofluene with concentrations around 2.9 and 1.6 mg 100 g^−1^ fw, respectively. The second main coloured carotenoid present in tomato is β-carotene, responsible for orangey colours. Its concentration is much lower, up to 1.2 mg 100 g^−1^ fw (Table 1). Apart from these major carotenoids, lesser amounts of γ-carotene, δ-carotene, lutein, neurosporene, α-carotene and other carotenoids can also be found in tomatoes [24,29] (Table 1).

Carotenoid distribution in the fruit is not regular. Lycopene can be found at higher concentration in the pericarp if compared with the locules, meanwhile β-carotene concentration is higher in the locules compared with the pericarp [25]. Moreover, lycopene concentration varies during the ripening process. Initially it starts to be present in the locules at the breaker stage, and then its concentration rises during the ripening process [30].

Polyphenols are present in tomato at lower concentrations (Table 1). These powerful antioxidants can be divided into different groups according to their core structure. Main tomato polyphenols are hydroxycinnamic acids, flavanones, flavonols, and anthocyanins. In addition, flavonol glycosides like rutin and kaempferol-3-rutinoside are also present in tomato fruits.

Naringenin chalcone is the main polyphenol found in tomato with concentrations up to 18.2 mg 100 g^−1^ fw [27]. The flavanone naringenin is present at lower concentrations, up to 1.3 mg 100 g^−1^ fw [26]. Quercetin is the main flavonol and one of the most important flavonoids from tomato. Its content varies from 0.7 to 4.4 mg 100 g^−1^ fw [26] in different tomato types. It can also be found in its glycosylated form as rutin, with concentrations up to 4.5 mg 100 g^−1^ fw [27]. The accumulation of rutin gives to the tomato peel its typical yellow colour. Chlorogenic acid is the main polyphenol from hydroxycinnamic acid family; its concentration ranges between 1.4 and 3.3 mg 100 g^−1^ fw [27]. Other flavonols such as kaempferol and myricetin are found in small quantities or traces in cultivated tomato, though they are present in related wild species [26,31,32].

The accumulation of flavonoids in tomato is tissue specific and develops at specific stages. For example, naringenin chalcone accumulates almost specifically in the peel simultaneously with the accumulation of carotenoids and degradation of chlorophylls, peaking up in overripe peels [33]. In fact, the flavonoid pathway is not active in the flesh due to the lack of expression of the genes involved in the flavonoid pathway [34]. Flavonol accumulation (mainly quercetin and kaempferol glucosides) is also almost (98%) restricted to the peel [35].

Usually the production of anthocyanins in tomato is restricted to vegetative tissues, such as the hypocotyl. Under certain conditions, including high irradiance and low temperature, they can accumulate in the stem and leaves. The main anthocyanins in tomato are derived from delphinidin, malvidin and petunidin [36,37].

Apart from an evident genotype and environmental effect on the accumulation of these bioactive compounds, in processed food their contents will also depend on the processing method, which may help to liberate compounds or may degrade them (reviewed by Nicoli *et al.* [38]). The nutritional value of tomatoes may be increased after thermal processing, as described by Dewanto *et al.* [39]. Their results pointed out that lycopene concentration is increased as it may be released from its natural matrix during the heat process. Dewanto *et al.* [39] also evaluated phenolic and total flavonoid content of heat-processed tomatoes, revealing non-significant changes in their content after the thermal process. Other authors have found a reduction of polyphenol content as a consequence of cooking. In this sense, Crozier *et al.* [40] showed an 82% of loss of quercetin content if tomatoes are boiled, a 65% loss if they are microwaved and 35% if they are fried. On the other hand, carotenoid concentration remains constant if tomatoes are boiled or even it can be more concentrated in tomato paste [41].

The results on the retention of polyphenols in tomato processed products are variable. Muir *et al.* [33] found that 65% of flavonols present in high-flavonol tomatoes were retained in processed tomato paste, while Stewart *et al.* [35] found that tomato flavonols like quercetin, can resist common processing methods and therefore they can be found in tomato-derived products such as tomato juice or tomato puree which are particularly rich these compounds. Obviating the relative differences in these studies, it seems clear that health benefits of processed tomato products may be similar or even higher than raw tomatoes, and make tomato-derived products a perfect alternative choice as functional food.

## 3. Chemoprotective Characteristics of Tomato Bioactive Compounds

### 3.1. Carotenoids

During the last several decades, many studies highlight the beneficial effects of the intake of tomato and tomato-derived products with a lower cancer risk (reviewed by Giovannucci [42]) and mainly with lower prostate cancer risk [43,44,45,46].

Considering the intake of tomato products, results from a 6-year follow-up cohort study by Mills *et al.* [47] pointed out a lesser prostate cancer risk associated to tomato consumption among other vegetables. In agreement to that, a meta-analysis carried out by Etminan *et al.* [45] highlighted a lower prostate cancer relative risk (RR) related to a high consumption of raw tomatoes (RR = 0.89, 95% CI) and specially cooked-tomato products (RR = 0.81, 95% CI). Giovannucci *et al.* [44] also reported a lower relative risk (RR = 0.77, 95% CI) from developing prostate cancer associated with the consumption of two servings of tomato sauce per week. In this study, a continuation of the Health Professionals Follow-Up Study (HPFS), the authors concluded that considering the moderate association found, it could be missed in a small studies or in those with substantial errors in measurement or based on a single dietary assessment. In a continuation of this study, Wu *et al.* [48] found a significant inverse association between higher plasma lycopene levels and lower risk of prostate cancer, though it was restricted to older men (age > 65) without a family history of prostate cancer (highest *vs.* lowest quintile Odds Ratio, OR = 0.43; 95% CI). At that point, few studies considered lycopene levels in the plasma. With a smaller sample, Lu *et al.* [49] found significant inverse associations between prostate cancer and lycopene plasma concentration (OR = 0.17, 95% CI). In this case, mean plasma lycopene level for the upper quartile was 401 mmol L^−1^. On the other hand, Kristal *et al.* [50] in the Prostate Cancer Prevention Trial (1683 cases and 1751 controls) analysed plasma lycopene levels (≥0.47 mg L^−1^ in the highest quartile) and found that prediagnostic serum lycopene concentration was not associated with the risk of total, low-, or high-grade prostate cancer incidence.

With the same cohort of the HPFS, in 2014 Zu *et al.* [51] pooled the results of 49,898 male health professionals from 1986 to 2010 and found a positive relationship between dietary lycopene intake and plasma lycopene levels, with mean plasma lycopene levels of 934.5 mol L^−1^ in the highest quintile of lycopene intake (n = 1200), and concluded that higher lycopene intake was inversely associated with total prostate cancer and more strongly with lethal prostate cancer. In fact, higher lycopene intake was associated with biomarkers in the cancer indicative of less angiogenic potential, thus probably reducing aggressive potential of cancer. The authors suggested that lycopene may inhibit angiogenesis of prostate cancer cells by regulating vascular endothelial growth factor, taking in consideration the results obtained by Yang *et al.* [52] in mice.

On the other hand, Kirsh *et al.* [53] in the Prostate, Lung, Colorectal, and Ovarian Cancer Screening Trial including 29,361 men during 4 years found no reason to support the hypothesis that higher tomato product consumption protects from prostate cancer. Indeed, the relation of lycopene intake and cancer has been controversial. As an example, in 2007 the US Food and Drug Administration concluded that there was limited evidence supporting an association between tomato consumption and reduced risk of prostate cancer [16]. But almost simultaneously, the World Cancer Research Fund and the American Institute for Cancer Research [54] concluded that foods containing lycopene may have a positive effect against prostate cancer. Interestingly, Zu *et al.* [51] suggested that lycopene intake would primarily affect prostate cancer progression.

The role of lycopene supplements in the evolution of cancer has also been analysed in clinical trials. In 2001 Kuckuk *et al.* [55] evaluated the impact of oral intake of lycopene (15 mg twice daily, during 3 weeks) in the evolution of patients with prostate cancer. In the intervention group, plasma prostate-specific antigen (PSA) levels decreased by 18% while in the control group they increased by 14%, and considering all biomarkers the authors concluded that lycopene supplementation may decrease the growth of prostate cancer, though the sample was too small to be conclusive.

Ansari and Gupta [56] found a 96% reduction of PSA levels after a 6-month supplementation of 2 mg lycopene twice daily in orchidectomy patients in comparison with patients only with surgical treatment (up to 90%). This study highlighted that the reduction turned to be more marked after two years of treatment (99% and 96% respectively). In other study with located prostate cancer patients, the serum PSA levels as well as leukocyte oxidative and prostate oxidative DNA damages were reduced after lycopene supplementation (30 mg lycopene per day during 3 weeks) before their radical prostatectomy [57]. Vaishampayan *et al.* [58] found that lycopene supplementation alone (30 mg day^−1^ in two doses) or combined with soy isoflavones (40 mg day^-1^ in two doses) had activity in prostate cancer patients with PSA relapse disease and may delay progression of both hormone-refractory and hormone-sensitive prostate cancer. Although the number of patients was limited (37 and 33 in each group). Other works, also highlighted the effects of lycopene delaying a high-grade prostate intraepithelial neoplastia (HGPIN) from developing into occult prostate cancer [59].

On the contrary, other authors have found no effect of lycopene supplementation and prostate cancer prevention or evolution. Regarding cancer evolution, Jatoi *et al.* [60] found no relationship between lycopene supplementation (30 mg day^−1^ during 16 months) and the progression of prostate cancer in 46 men with androgen-independent prostate cancer. Clark *et al.* [61] tested wider dose ranges of lycopene supplementation (15–120 mg day^−1^) in 36 patients with biochemical relapse of prostate cancer, and found no stabilization of PSA levels as a consequence of the treatment. They also found that plasma lycopene levels were similar for the 15–90 mg day^−1^ doses when the concentration reached a plateau after three months of supplementation. Bunker *et al.* [62] studied the evolution of 81 men with high-grade prostatic intraepithelial neoplasia, atypical foci or repeated non-cancerous biopsies and found no effect of lycopene supplementation (30 mg day^−1^) after the first month of treatment. But the same authors suggested that lowering of serum PSA may not be an appropriate endpoint for the long-term studies on the effect of lycopene supplementation for reducing prostate cancer initiation or progression.

Prostate cancer preventing properties of other tomato carotenoids have also been questioned in other works. In that sense, Neuhouser *et al.* [63] revealed in the Beta-Carotene and Retinol Efficacy Trial (CARET) that the supplementation with β-carotene (30 mg day^−1^) and retinol (25,000 IU retinyl palmitate day^−1^) had no significant effect on total prostate cancer incidence. In fact, it could be negative, as they found that supplemented individuals had higher relative risk (RR = 1.52, 95% CI) of developing an aggressive prostate cancer (Gleason score 7–10) that was reduced in the post-intervention period (RR = 0.75, 95% CI). CARET only included smokers, thus this results may not apply to non-smokers.

Apart from prostate cancer, the incidence of other cancers has been also associated with tomato carotenoids. In 2007, the World Cancer Research Fund and the American Institute for Cancer Research [54] concluded that apart from the positive effects of lycopene containing foods on prostate cancer, foods containing carotenoids probably protected against cancers of the mouth, pharynx, and larynx, and also lung cancer, and specifically, those containing β-carotene would probably protect against oesophageal cancer. In this sense, the meta-analysis performed by Ge *et al.* [64] confirmed that higher intake of β-carotene, α-carotene, lycopene, β-cryptoxanthin, lutein, and zeaxanthin reduced oesophageal cancer risk with pooled ORs of 0.58, 0.81, 0.75, 0.80, and 0.71 (95% CI), respectively.

Regarding lung cancer, in 1996 Ziegler *et al.* [65] concluded that β-carotene may have a role in the prevention of lung cancer, but the dosage should be carefully studied, as lung cancer incidence and mortality were increased in studies with β-carotene supplementation with male smokers. In fact, results from Alpha-Tocopherol Beta-Carotene (ATBC) study revealed higher lung cancer incidence in participants who received β-carotene supplementation, 20 mg day^−1^ during 8 years [66], especially among smokers [67]. Similarly, the CARET study highlighted that β-carotene supplementation had an adverse effect on lung cancer incidence and lung cancer mortality in smokers and workers exposed to asbestos [68]. It has been proposed that this procarcinogenic effect may be explained by the activity of β-carotene-oxidized products produced as a consequence of the free radical-rich environment in the lungs of cigarette smokers [69]. Wright *et al.* [70] suggested that the increased risk of lung cancer would be related to aberrant cell growth, while Mondul *et al.* [71] found that the metabolomic profiles of response to β-carotene supplementation pointed to the induction of cytochrome p450 enzymes CYP1A2 and CYP2E1. As several medications used for cardiovascular diseases are metabolized by CYP1A2, an interaction between the supplemented β-carotene and prescribed medication may have resulted in the increased mortality found in the ATBC study, though a dysregulated glycemic control could also have contributed. These findings stressed the importance to increase our knowledge on the possible interactions between dietary supplements and other pharmacological agents. Nevertheless, the fact that supplementation at high doses may have adverse effects, does not exclude a positive effect at dietary levels [72]. Other works have found no effect of β-carotene supplementation and lung cancer incidence [73].

Controversy also applies in the case of breast cancer. Eliassen *et al.* [74] carried out a pooled analysis of eight cohort studies where serum carotenoids and breast cancer risk were evaluated. This study pointed out a reduced relative risk of suffering breast cancer among women with higher plasma carotenoids. Among tomato carotenoids, the authors found a lower relative risk for lycopene (RR = 0.78, 95% CI) followed by β-carotene (RR = 0.83, 95% CI). Pantavos *et al.* [75] analysing dietary intake of antioxidants in  3209 women found that low intake of α-carotene and β-carotene was associated with a higher risk of breast cancer among smokers (Hazard Ratio, HR = 2.48, 95% CI and HR = 2.31, 95% CI for α- and β-carotene, respectively). On the other hand, Greenlee *et al.* [76] studying the Life After Cancer Epidemiology (LACE) cohort (2264 women diagnosed of breast cancer) concluded that frequent use of combination carotenoids was associated with increased risk of death from breast cancer (HR = 2.07, 95% CI) and all-cause mortality (HR = 1.75, 95% CI).

Results from the Linxian Cancer Prevention Study highlighted a significant reduction in cancer mortality (RR = 0.87, 95% CI), especially from stomach cancer (RR = 0.79, 95% CI) after β-carotene, vitamin E and selenium supplementation [77]. But a recent meta-analysis [78] concluded that although data from case-control studies suggested a protective role of α- and β-carotene against gastric cancer, there were inconsistencies compared with cohort studies, and no conclusive evidence was found for this suggestion.

The mechanism of action of carotenoids in cancer prevention was extensively reviewed by Tanaka *et al.* [72] and Trejo-Solís *et al.* [79]. Antioxidant capacity of these compounds may justify part of this protection. Carotenoids exert strong antioxidant capacity because they contain many double-conjugated bonds. In that sense, lycopene can act as antioxidant because its ability to trap the ^1^O_2_ twice as effective as that of β-carotene. Moreover, another antioxidant potential of lycopene is to react with free radicals. The elimination of these Reactive Oxygen Species (ROS) is important in the cancer chemoprevention since they can facilitate the carcinogenesis-related processes by means of the oxidation of cellular biomolecules. In addition, lycopene may exert its effect though the regulation of the antioxidant response element (reviewed by Trejo-Solís *et al.* [79]).

But apart from their antioxidant capacity, other mechanisms contribute to cancer prevention, including immune modulation, hormone and growth factor signaling, regulatory mechanisms of cell cycle progression, cell differentiation and apoptosis. In fact, the existence of this variety of mechanisms has led to propose that the initial effect of carotenoids should involve modulation of transcription (reviewed by Tanaka *et al.* [72]).

### 3.2. Polyphenols

Plant polyphenols have been reported to interfere with the initiation, promotion and progression of cancer [80]. In fact, polyphenols exert numerous effects on tumorigenic cell transformation, on tumour cells *in vitro* and *in vivo*, and may interact with conventional anti-tumour therapies [81]. There exist different polyphenol families and they differ in their chemoprotective capabilities. For example, among 68 polyphenols, the order of their potency to suppress *in vitro* the human liver cancer cells resulted to be chalcones > flavones > chromones > isoflavones > flavanones > coumarins [82].

The effect of the different classes of polyphenols on cancer risk incidence has been evaluated in several case-control studies [83,84,85,86]. In that line, Bosetti *et al.* [85] evaluated the intake of different families of polyphenols and their effect on women breast cancer incidence in a large case-control study carried out in Italy during 4 years. A reduced risk of suffering breast cancer was related with a high dietary intake of flavonols (>29.9 mg day^−1^) (highest *vs.* lowest quintile OR = 0.80, 95% CI) and flavones (>0.6 mg day^−1^) (OR = 0.81, 95% CI), but no significant associations were found for other tomato polyphenols such as flavanones (>62.2 mg day^−1^) (OR = 0.95, 95% CI) and anthocyanidins (>20.5 mg day^−1^) (OR = 1.09, 95% CI). Specifically, a diet rich in quercetin (4.7 mg day^−1^) have also been associated with a lower breast cancer risk in women (RR = 0.62, 95% CI) [87]. On the other hand, another case-control study carried out in Greece evaluating fruit and vegetable consumption found no associations between dietary intake of flavonols, flavanones, flavan-3-ols, isoflavones or anthocyandins and breast cancer risk; however, a statistically lower risk (OR = 0.87, 95% CI) were found for flavone intake (0.5 mg day^−1^) [84].

Phenolic compounds also seem to protect against colorectal cancer. In this case, a large case-control study carried out by Rossi *et al.* [86] in Italy showed that diets with a higher intake of flavonols (>28.5 mg day^−1^) (OR = 0.64, 95% CI) and anthocyanidins (>31.7 mg day^−1^) (OR = 0.67, 95% CI) among other phenolic compounds were associated with a lower risk of suffering colorectal cancer. However, the same study found no associations between total flavonoids and flavanones and colorectal cancer. Other studies went further and tested the dose-response activity of quercetin. In that line, Cruz-Correa *et al.* [88] found that a joint supplementation of 20 mg quercetin together with 480 mg curcumin thrice per day prior colectomy were capable to decrease the number and size (60.4% and 50.9% from baseline respectively) of ileal and rectal adenomas in patients with familial adenomatous polyposis (FAP).

Flavonols also would exert a protective effect against stomach cancer. García-Closas *et al.* [83] evaluating food habits in Spanish participants found a lower risk of suffering stomach cancer in participants with higher dietary intakes of quercetin (>8.5 mg day^−1^) (OR = 0.62, 95% CI). Other flavonols like kaempferol and myricetin were also evaluated in this work, but the authors found that only kaempferol (dietary intakes higher than 1.3 mg day^−1^) also had protective effects (OR = 0.48, 95% CI). Some studies though have discarded a significant protective affect against these types of cancer [87].

The chemopreventive effects of polyphenols against lung cancer have also been proven (reviewed by Neuhouser [89]). Among them, the results from the Finnish Mobile Clinic Health Examination Survey pointed that these chemoprotective effects of dietary flavonoid intake (3.51 mg day^−1^) (RR = 0.54, 95% CI) may be mainly attributed to quercetin [90]. Knekt *et al.* [87] evaluated the dietary intakes of participants in basis of flavonoid concentration in the same study, reporting a lower incidence of total cancer associated with higher intakes of quercetin (RR = 0.77, 95% CI), especially for lung cancer in men (3.9 mg day^−1^) (RR = 0.42, 95% CI). The cohort study carried out by Hirvonen *et al.* [91] also revealed an inverse relationship between the joint dietary intake of flavones and flavonols (16.3 mg day^−1^) and lung cancer risk (RR = 0.56, 95% CI) in male smokers from the ATBC study.

Regarding liver cancer, Lagiou *et al.* [92] also found a chemoprotective effect of dietary flavone intake (>1.16 mg day^−1^) and hepatocellular carcinoma (OR = 0.41, 95% CI) for patients without hepatitis. An inverse association was also found between dietary consumption of flavan-3-ol (>66.3 mg day^−1^), anthocyanidins (>152.7 mg day^−1^) and total flavonoids (>358.1 mg day^−1^) and cholangiocarcinoma (though only few cases were available for the study).

Anti-cancer properties of other tomato polyphenols such as chlorogenic and caffeic acids have been less studied in patients; however there are many studies where they have been tested using cellular models (reviewed by Weng and Yen [93]). In that line, Yang *et al.* [94] found that chlorogenic acid may induce apoptosis *in vitro* in human leukemia U937 cells by the reduction of the levels of mitochondrial membrane potential and increasing the activation of caspase-3-pathways. The effect of caffeic acid on fibrosarcoma HT-1080 cell line was evaluated by Rajendra-Prasad *et al.* [95]. Their results pointed out that caffeic acid treatments can lower the cell viability of fibrosarcoma cells. Other tomato phenolic compounds like naringenin present anti-cancer properties *in vitro*. In that line, some works pointed out the anti-proliferative effects of naringenin in different cancer cell lines like HT29 colon cancer cells [96] or MCF-7 breast cancer cells [97]. Moreover, combinations of different polyphenols have been also evaluated revealing that naringenin and quercetin are effective inhibitors of the MDA-MB-435 human breast carcinoma cell line [98].

The ability of phenolic compounds as reactive oxygen scavengers is the main motivator of its antioxidant properties. Among the main polyphenols present in vegetables, the action of quercetin in *in vivo* studies seems to be related with the inhibition and induction of survival and death signalling pathways respectively in liver cancer cells and its strong antioxidant activity and consequent prevention of ROS-induced DNA mutations in critical genes for cell cycle control [99]. In addition to ROS quenching, the antioxidant protective effect of flavonoids may be related to their modulating activity of several detoxifying enzymes like lipoxygenase, cyclooxygenase, inducible nitric oxide synthase, monooxygenase, xanthine oxidase and NADH oxidase (reviewed by Gibellini *et al.* [100]). Moreover, quercetin also can act in the chromatin remodelling and thus interfering with epigenetic alterations which are important in cancer progression. The effect of polyphenols on phase-I and -II enzymes can also modulate procarcinogenic metabolism. The modulation of NF-κB molecular pathway is another mechanism of polyphenol chemoprevention properties. In that sense, caffeic acid would inhibit growth and metastasis of HCC through modulation of expression of proteins involved mainly in NF-κB molecular pathway. Anyway, more research should be done in this field due to the vast number of potential antioxidant compounds from the polyphenol family.

## 4. Breeding Strategies for the Improvement of Carotenoid and Polyphenol Content in Tomato

Improvement of functional quality is a complex task because the accumulation of any antioxidant compound is the result of complex metabolic processes. Consequently, the increase in the available information on antioxidant biosynthetic pathways (enzymes involved and regulation mechanisms) and the identification of mutant genotypes with beneficial pathway alterations for the antioxidant accumulation is essential to obtain higher precision and better results in the development of breeding programs. Different breeding strategies can be followed with this purpose, and they all depend on the existence of variability for the accumulation of bioactive compounds in the cultivated species or wild relatives. Additionally, a different approach can be followed via genetic engineering. Advances in the improvement of carotenoid and polyphenol composition of tomato are reviewed herein.

### 4.1. Carotenoids

**Figure 1:**
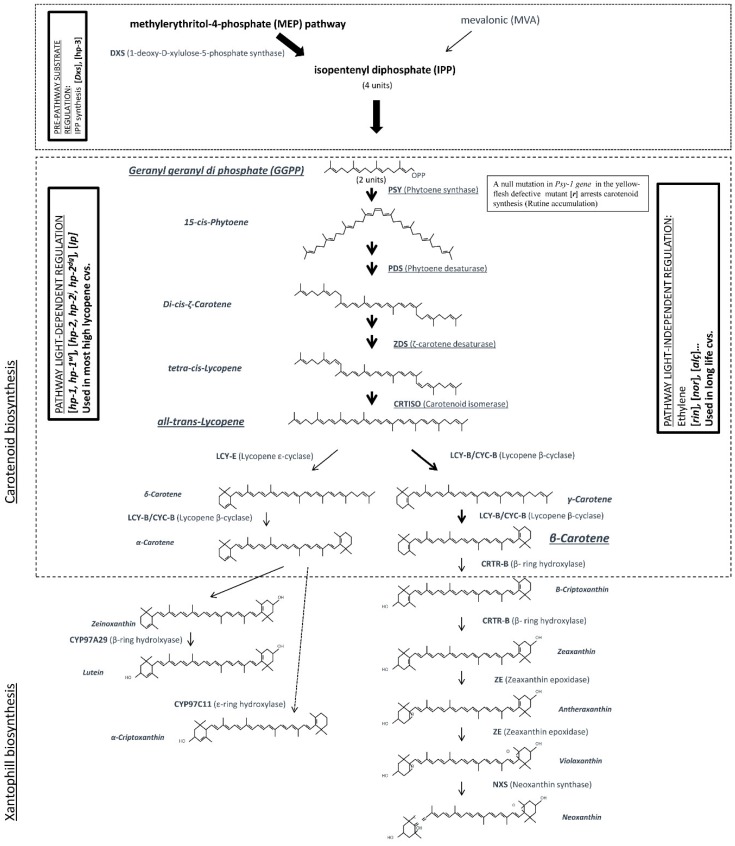
Carotenoid and xanthophyll biosynthesis pathway in tomato (adapted from; [24,110,112,122,123]). The most important products and steps in carotenoid biosynthesis in common red tomatoes are underlined and marked with thick arrows respectively. The three regulation mechanisms are indicated with 90° rotated boxes.

In tomato, breeding for fruit colour was one of the first fruit quality objectives demanded by markets. As this feature is conferred by carotenoid pigments (mainly lycopene and β-carotene) with important antioxidant properties, the improvement of fruit colour has indirectly led to the improvement of tomato nutritional and functional value. This situation justifies that breeding for improved carotenoid content is far more advanced than any other bioactive compounds. The high colour variability present in genus *Solanum* section *Lycopersicum* allowed the first studies of diverse genetic variants (natural mutants) for this attribute (Table 2). Studies on tomato natural mutants for carotenoid accumulation and on tomato transgenic plants with different carotenoid biosynthetic pathway alterations has allowed a detailed knowledge of the complete tomato carotenoid biosynthesis pathway (Figure 1) and its regulation strategies.

In common red tomatoes, from the start of the maturing process (breaker stage) a high increment in synthesis of the enzymes PSY [101], PDS [102] and CRTISO [103] occurs, which results in a high increment in the all-trans-lycopene synthesis (marked with thickness arrows in Figure 1). At the same time, a strong repression of the synthesis of lycopene cyclases (enzymes involved in the formation of 6C cyclic end groups) occurs [104]. Usually, in common red tomatoes, only lycopene-β-cyclases, LCY-B [102] and the chromoplast specific CYC-B [105] enzymes are present. The drastic diminution of the lycopene-β-cyclases entails that only a low amount of β-carotene synthesis at the expense of lycopene can be achieved (Figure 1). Consequently, the prominent carotenoid in mature fruits will be all-*trans*-lycopene, which confers to the ripe fruits its typical red colour. Several natural mutant genes with altered steps in this biosynthetic pathway have been identified (Table 2). As a result, altered fruit carotenoid profile and fruit colours are obtained [103,105,106,107]. In green tissues of the plant, the carotenoid biosynthesis pathway does not stop with lycopene accumulation, but it continues with the xanthophyll biosynthesis pathway (Figure 1) and neoxanthin would be the last product synthesized, deriving in the abscisic acid (ABA) synthesis pathway [24].

Regulation of the carotenoid biosynthesis process can be done at three different levels. The first level consists in the regulation of the initial amount of the precursor of the synthesis of carotenoids (pre-pathway substrate regulation, Figure 1), the isopentenyl diphosphate (IPP), which determines the total amount of carotenoids that can be synthesised. IPP may arise at some developmental stages partly from the citoplasmic mevalonic (MVA) pathway [108], but it is mainly synthesised through the methylerythritol-4-phosphate (MEP) pathway, apparently bound to the plastidic compartment [109]. The first enzyme of MEP pathway, 1-deoxy-D-xylulose-5-phosphate synthase (DXS), has been proved to catalyse the first regulatory step in carotenoid biosynthesis [110] and, consequently, highly influences the final amount of carotenoids. This pre-pathway substrate regulation also can be altered by a reduction of ABA synthesis which has as consequence an increase of plastid division enabling higher biosynthesis and accumulation of carotenoids [111]. This was observed in high pigment-3 (hp-3) mutants. These mutants coding a defective zeaxanthin epoxidase (ZE) enzyme (Xanthophyll biosynthesis pathway, Figure 1) which arrest ABA synthesis and, as explained above, increased carotenoid accumulation.

A second level of regulation consists in the hormonal growth regulation of the fruit ripening process (light independent regulation, Figure 1) which also plays a major role in the control of the described carotenoid synthesis pathway. During the ripening process, ethylene has a strong positive control of the increment in the mRNA levels for the lycopene-producing enzymes phytoene synthase (PSY) and phytoene desaturase (PDS), at the same time, the mRNA levels of the genes for the lycopene β- and ε-cyclases diminish and completely disappear [104]. This explains that mutations affecting ethylene synthesis or perception, such as long life mutations *rin*, *nor*, *alç*..., not only delay the normal ripening process also result in altered carotenoid content [112].

Finally, there is a third level of regulation in which fruit localized phytochromes also regulate the extent of carotenoid accumulation [113]. Phytochrome response to light presence enables carotenoid synthesis that stops in darkness, thus conditioning day-cyclical biosynthesis periods (light-dependent regulation box, Figure 1). Several natural mutants present alterations in the normal phytochrome regulation with enhanced global carotenoid content (Table 2): the mutation *high pigment-1* (*hp-1*), and the allelic *hp-1^w^* results in the plant acting as perceiving continuously the light [114]. The mutation *high pigment-2* (*hp-2*), and the allelic *hp-2^j^* and *hp-2^dg^* [115], affects the photomorphogenesis regulatory gene TDET1, and also affects the light signal-transduction machinery [116]. Another mutant, Intense pigment (*Ip*) is implicated in a promotion of phytochrome signal amplification [117,118].

All the breeding strategies targeted to improve carotenoid content in tomato try to achieve a gene combination enabling a higher accumulation of one or more carotenoids in a desired genotype with good agronomic performance. Two main strategies can be used to do it: one is the use of germplasm with a high potential to accumulate one or more antioxidants as a gene donor in order to transfer them by conventional breeding programs and the other the use of advanced biotechnology to transfer foreign genes (genetic engineering) to allow a beneficial biosynthesis alteration of the desired antioxidant compound.

Both strategies can be complementary, and in fact genetic engineering can be used to avoid the negative side effects of certain genes changing their promotor (reviewed by Cebolla-Cornejo *et al.* [112]). Nevertheless, the level of public scepticism in certain regions (e.g., Europe) hinders the commercialization of transgenic varieties [3] and commercial approaches are right now based in conventional breeding programs.

In summary, the first approach involved the exploitation of natural diversity present in the genus (mainly genotypes with natural gene mutations identified in many studies and described before) as their use as donor parents in conventional breeding programs following different hybridization strategies and selection generations. Following this approach, some of the more successful fresh tomato cultivars developed carrying the Beta, B, gene with its modifier Beta-modifier, moB (Figure 1) have been Caro Red [119] and Caro Rich [120] and the processing tomato cultivar Caro beta [121]. These cultivars show orange fruits with β-carotene contents up to 5 mg 100 g^−1^ fw (roughly up to 10-fold the normal content in standard red cultivars). Unfortunately, these orange fruited cultivars with high β-carotene content have not been commercially successful, as consumers seem to prefer red tomato fruits.

Other approaches focused in the increase of lycopene content have obtained improved cultivars using mutants *old gold* (*og*) and *old gold crimson* (*og^c^*) which inhibits the synthesis of β-carotene by cyclisation of lycopene resulting in higher lycopene contents (up to 30% of normal content) and lower β-carotene accumulations [122]. Tomato cultivars of the crimson type have been successfully commercialized due to their intense red pigmentation even when the fruits are not completely ripe. One of the problems of the use of mutants affecting single steps of the biosynthesis pathway is that the increase in the level of one carotenoid is obtained at the expense of another, thus little effect can be expected for total carotenoid content. Thus, mutants affecting the regulation of the pathway would have a more dramatic effect. In fact, better results have been obtained with cultivars carrying both crimson and high pigment genes (*hp-1* or *hp-2* alleles), as increments in the lycopene content up to 3- to 4-fold of common cultivars has been obtained [124]. Although, some deleterious effects on seed germination, plant vigour and yield have been associated with the high pigment mutants. The best expectations where deposited in the use of *Intense pigment* (*Ip*) mutant gene. This gene allows carotenoid accumulation similar to those of *hp* genes, but with lower deleterious effects and it is dominant (commercial hybrid development more interesting than with recessive genes as *hp* mutants) [125].

Regarding the second breeding strategy, the high advances in the knowledge of the carotenoid biosynthesis (metabolic pathway, precursors and regulation mechanisms) allowed the use of this information to obtain several experimental transgenic tomato lines with modified genes controlling some biosynthetic steeps. Most of this works try to emulate the carotenoid biosynthetic performance of some of the natural mutants identified, and some of them also try to avoid undesirable side-effects found in these mutants.

One approach used has been the modification of isoprenoid precursor’s pathway (pre-pathway substrate regulation) intervening in the both mevalonate (MVA; [109]) and methylerythritol-4-phosphate (MEP; [126]) pathways (Figure 1) trying to increase the total amount of carotenoids increasing the levels of the precursor. Only transformation with gene encoding 1-deoxy-D-xylulose-5-phosphate synthase (DXS) from *Escherichia coli* to increase MEP pathway showed interesting results (2.2 times higher β-carotene content).

The most explored approach has been placed in the development of transgenic lines with altered expression of the most important enzymes in the carotenoid synthetic pathway with the objective to alter the carotenoid profile and to increase the content of certain of interesting carotenoids. The focus was put on the enzymes phytoene synthase [127,128], phytoene desaturase [129] and mainly on lycopene cyclases [105,130,131,132,133,134] due to their role in the regulation of the whole pathway and its role in the partition of the main carotenoids lycopene and β-carotene. Several strategies have been used: constitutive or selective expression of foreign genes from several species and overexpression or repression of target genes by several mechanisms, but results obtained were contradictory. To avoid these problems, in recent years, a more refined strategy targeted to obtain a better expression of transgenes using more selective and specific promoters have been explored. In this sense, the characterization of the tomato PDS promoter [135], as well as *S. habrochaites* lycopene β-cyclase (CYC-B) promoter [136] bring information to enhance the use of new transgenes. Other efforts were targeted to use promoters which allows a selective expression of transgenes in fruit tissues and adequate developmental stages as occurs with fruit specific promoters such as ethylene response genes E8 and E4m polygalacturonase and lipoxygenase, mainly acting in the late-ripening stage or the LA22CD07 and LesAffx.6852.1.Sl at in green and red-ripening fruits [137].

Finally, the third approach used is based on the modification of some of the regulatory mechanisms of the carotenoid biosynthesis pathway. In this sense, some transgenic experimental lines tried to emulate the performance of high pigments mutants *hp-1* [114] and *hp-2* [138]. In this last case, the strategy used involved the suppression of photomorphogenesis regulatory gene TDET1 in fruits (using fruit specific promoters combined with a RNA interference approach based in inverted repeat constructs) to reduce negative collateral vegetative effects, and gave interesting results. Other works were focused in the development of transgenic experimental lines simulating performance of *hp-3* mutants. In this sense, the down-regulation of ABA synthesis during ripening using an RNAi construct of the *SINCED1* gene driven by the fruit specific promoter E8 [139,140], caused a reduction in ABA concentration, an increase of ethylene production and resulted in an increment in lycopene and β-carotene contents.

### 4.2. Polyphenols

The polyphenol content of tomatoes has gained importance during the last decade. Consequently, the achievements of breeding efforts still lag behind those obtained for carotenoids. Nevertheless, quite a lot of information is available regarding polyphenol biosynthesis in this crop. In fact, the polyphenol metabolic pathway has also been ascertained. Several transcription factors related with the regulation of polyphenol biosynthesis have been identified, but a lot of information regarding the spatial accumulation of polyphenols in the fruit and how it can be reverted is still required.

**Figure 2:**
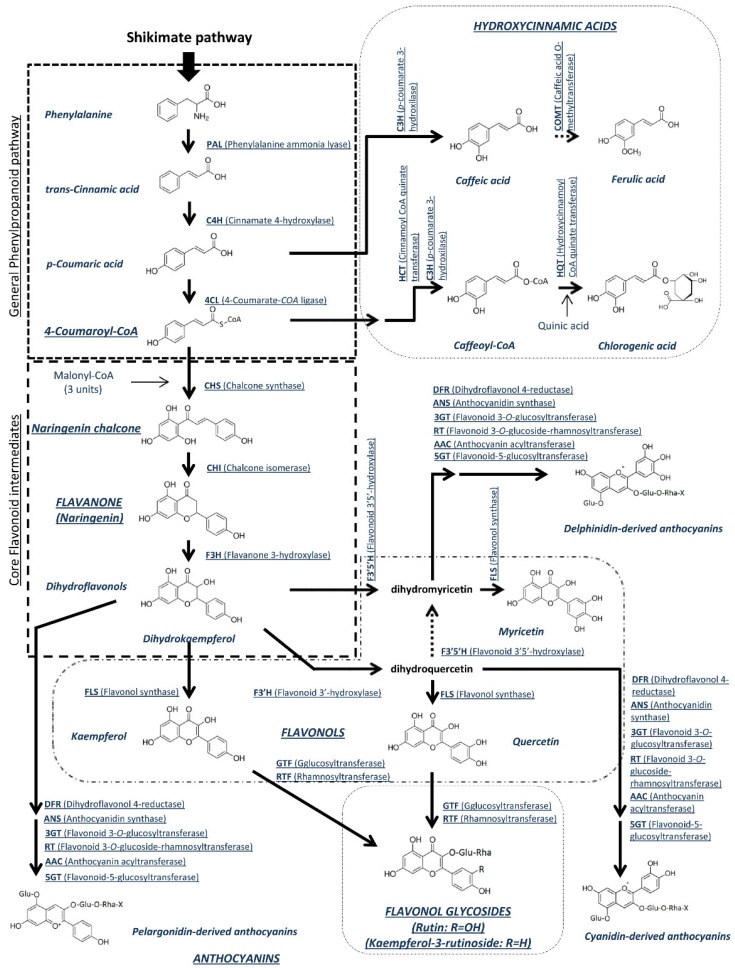
Polyphenol biosynthesis pathway in tomato (adapted from [141,142,145,146,147,148]. Broken arrows indicate common pathways in plants, which would probably apply to tomato. Solid lines confirmed in tomato.

The phenylpropanoid biosynthetic pathway is the first step in the polyphenol biosynthesis and it uses the amino acid phenylalanine from the shikimate pathway as initial substrate (Figure 2). This biosynthetic pathway is common for two of the main classes of tomato polyphenols: hydroxycinnamic acids and flavonoids. The first step involves the conversion of phenylalanine into *trans*-cinnamic acid using the enzyme phenylalanine ammonia lyase (PAL). The enzyme cinnamate 4-hydroxylase (C4H) catalyses the conversion of the resulting product into *p*-coumaric acid. At this point, the flavonoid biosynthetic pathway continues with the conversion of *p*-coumaric acid into 4-coumaroyl-*CoA* as a result of the action of 4-coumarate-CoA ligase (4CL) [141].

Meanwhile the hydroxycinnamic acid pathway continues with the transformation of *p*-coumaric acid into caffeic acid catalysed by the p-coumarate 3-hydroxylase (C3H) [142]. Chlorogenic acid, one of the main polyphenols present in tomato [26], is formed from caffeoyl-CoA, which is transesterificated with quinic acid by hydroxycinnamoyl-Coenzyme A:quinate hydroxycinnamoyl transferase, HQT [143]. Caffeoyl-CoA would be obtained from 4-coumaroyl-CoA in three steps involving the successive activities of cinnamoyl CoA shikimate/quinate transferase (HCT), *p*-coumaroyl ester 3-hydroxylase (C3H) and HCT [144]. Finally, the third main hydroxycinnamic acid in tomato, ferulic acid, would be obtained from caffeic acid with the enzyme caffeic acid *O*-methyltransferase (COMT) [142].

On the other hand, the core flavonoid biosynthetic pathway starts with the conversion of the resulting 4-coumaroyl-*CoA* from the phenylpropanoid pathway into the yellow-coloured naringenin chalcone [141]. This key reaction is performed by the enzyme chalcone synthase (CHS) which begins with the condensation of one molecule of 4-coumaroyl-*CoA* with three molecules of malonyl-*CoA*. In most plants, including tomato, chalcones are not the end-product of the pathway. The enzyme chalcone isomerase (CHI) isomerizes naringenin chalcone into the flavanone naringenin. Finally, the core flavonoid intermediates pathway finishes with the formation of the dihydroflavonol dihydrokaempferol as a result of the action of the flavanone 3-hydroxylase (F3H). From this central intermediate, the flavonoid biosynthetic pathway diverges into several side branches, each resulting in a different class of flavonoid [141].

One of the most important classes of flavonoids in tomato are flavonols [26,149], which are synthesised from dihydrokaempferol. The three more important in tomato are kaempferol, quercetin, and myricetin. The three are formed from the corresponding dihydroflavonol, and have dihydrokaempferol as a starting point.

Flavonol synthase (FLS) catalyses the direct conversion of dihydrokaempferol into kaempferol. On the other hand, with dihydrokaempferol as substrate, the enzymes flavonoid 3’-hydroxylase (F3’H) and FLS would produce respectively dihydroquercetin and quercetin [147]. Quercetin is especially important in tomato, as it is the base for the formation of rutin. This quercetin glycoside is quite abundant in tomato [32], and it is obtained from quercetin by the action of the enzymes glucosyltransferase (GTF) and rhamnosyltransferase (RTF) [146].

The third flavonol, myricetin, would be derived in from dihydroquercetin in two steps. The first one catalysed by flavonoid 3’5’-hydroxylase, F3’5’H [145], would produce dihydromyricentin which would be converted into myricetin by FLS. Moreover, myricetin could also be directly obtained from dihydrokaempferol by means of the action of F3’5’H and FLS [145,146,147].

Anthocyanins are not naturally accumulated in the tomato fruit, but as it will be shown, interspecific crosses can restore this pathway in the fruit. Their synthesis would start from the three commented dihydroflavonols. Dihydroflavonol 4-reductase (DFR) would catalyse the conversion of dihydroflavonols into flavan-3,4-diols, which would be then transformed into anthocyanidins by anthocyanidin synthase (ANS). The final step would require the addition of sugars to form anthocyanins, which are anthocyanidin glycosides [141,144]. The main anthocyanins present in tomato would be derived from three anthocyanidins: delphidin, that would be synthesised following this scheme from dihydromyricetin, cyaniding from dihydroquercetin and pelargonidin from dihydrokaempferol. Other anthocyanidins would be derived from these. For example, delphinidin can be methylated on its 3’ hydroxyl group to form petunidin or on both its 3’ and 5’ hydroxyl groups to form malvidin [150].

As in the case of carotenoids, several mutants have been identified regarding polyphenol accumulation (Table 3), and those more used in breeding programs are related to the regulation of the pathway. It seems that CHI plays a central role in the rate determining step in the production of flavonols [151]. In fact, its over-expression increases dramatically the levels of flavonols at the expense of naringenin chalcone [33]. Ballester *et al.* [152] suggested that upon ripening an increase in the expression CHS expression and a coordinated decrease in the expression of CHI would result in the accumulation of naringenin chalcone and in a limitation of flavonol contents. Previously, Willits *et al.* [153] suggested that the lack of expression of CHI in the peel of the fruit, probably caused by a mutation in a fruit specific promoter would explain the high levels of naringenin chalcone in this tissue. The authors also assumed that cultivated tomato would have lost the expression of CHI in the peel in an early step of the domestication process.

Naringenin chalcone is one of the prominent polyphenols in tomato. Its accumulation in the peel gives a yellow colour that in combination with red flesh results in an external red colour of the fruit. Several tomato landraces are characterized by an external pink colour, resulting from the lack of accumulation of naringenin chalcone and a consequent transparent peel. The evaluation of introgression lines of *Solanum chmielewskii* in tomato enabled the identification of the gene responsible for this mutation (*yellow*, *y*), initially described in 1925 [154]. This gene encodes SlMYB12, a transcription factor involved in the regulation of the phenylpropanoid and/or flavonoid pathway [152].

The expression of the *Arabidopsis* form AtMYB12 in tomato induced primary and secondary metabolism, binding directly to the promoters of different genes encoding enzymes of the primary metabolism such as 3-deoxy-D-arabino-heptulosonate 7-phosphate synthase, DAHPS, and plastidial enolase, ENO [155]. DAHPS is a key determinant if the flow of the shikimate pathway. A first step needed for the accumulation of phenylalanine, a substrate necessary for the flavonoid pathway. Additionally, it would also bind to promoters of genes related enzymes participating in the flavonoid pathway (PAL5A, PAL5C, PAL5D, CHS1 and F3H). As result a dramatic increase in flavonol and hydroxycinnamates is observed, reaching up to 10% of fruit dry weight.

Pandey *et al.* [156] also expressed constitutively AtMYB15, obtaining similar results. In this case, 305 unigenes were upregulated in the fruit tissue and 419 downregulated. Specifically, several enzymes of the flavonoid pathway were upregulated, especially CHS (300-fold enhanced expression in the fruit) and FLS (300-fold enhanced expression in the fruit). Additionally, genes involved in ethylene biosynthesis and signalling, ABA, auxin and Ga signalling were also modulated. Primary metabolism was also altered, and a differential regulation of genes involved in aromatic amino acid biosynthesis and carbohydrate metabolism was observed. Probably this modulation may be related to the elevated demand of C-source to support the enhanced biosynthesis of polyphenols.

Recent transcriptome analysis has identified additional transcription factors involved in the regulation of the pathway [157]. At least 20 transcription factors would correlate with expression of genes participating in the flavonoid biosynthesis pathway. As expected, SlMYB12 is included in this set. Other examples include LIM, which is highly correlated with the expression of genes involved in both the biosynthesis of ascorbic acid and flavonoids, and other MYB and bHLH genes.

The expression of endogenous genes encoding key enzymes of the flavonoid pathway (PAL, CHS, CHI, F3H and FLS) in tomato pericarp and collumela tissues has been found to be under detection limits [146]. This lack of expression would explain the low amounts of flavonoids found in these tissues. The expression of these genes can be achieved. For example, an overall increase in flavonol accumulation in the whole fruit was achieved with the expression Lc and C1 transcription factors from maize [151]. In this case, a clear over-expression of genes encoding CHS and F3H was observed, suggesting that the expression of these two genes would be necessary to improve flavonoid accumulation in tomato flesh. Other studies confirmed that the expression of the gene encoding FLS would also be required alter all [34]. In fact, the joint over-expression of genes encoding CHS and FLS would be required to pull the carbon flux towards the accumulation of flavonols [146].

Regarding the light regulation of the pathway, Giuntini *et al.* [158] showed that UV-B radiation differentially affects the expression of genes of the flavonoid pathway and results in altered content of flavonoids and hydroxycinnamic acids. The conventional cultivar Esperanza showed higher levels of naringenin chalcone, quercetin and rutin and of sinapic, caffeic, ferulic and *p*-coumaric acids in the flesh of UV-B shielded fruits at the red ripe stage, while in the high lycopene cultivar DRW5981 limited effects were observed at this stage, especially for flavonoid content. In the conventional cultivar Esperanza, the expression of genes encoding CHS and CHI expression was higher and that corresponding to F3H and F3’H was reduced in UV-B shielded fruits. In both cultivars, carotenoid accumulation followed the same response of flavonoid accumulation. In the high lycopene cultivar DRW5981 several genes of the flavonoid pathway were upregulated in the fruit flesh with the exception of *CHI*. As a result a dramatic increase in naringenin chalcone and a modest increase in quercetin were observed.

As commented in the carotenoid section, the *high pigment* (*hp*) tomato mutants also affect the light regulation of flavonoid synthesis. This overproduction of bioactive compounds is also associated with increased plastid biogenesis and therefore plastid-accumulating metabolites would be expected (reviewed by Azari *et al.* [159]).

The increase in the accumulation of flavonoids in conventional breeding programs, excluding genetic engineering has offered limited results. In fact, the analysis of different tomato germplasm for flavonol accumulation offered limited levels of variation (up to 10-fold). Quercetin in hydrolysed peel extracts varied between 6.3 and 64.9 μg g^−1^ fw, but the highest levels only represented a 2.5-fold increase compared to a standard commercial variety [34]. Similar levels of variation have been found by other authors, with the highest levels been found in the smaller cherry tomato fruits originating from sunny climates [35]. The limited variation found in the cultivated tomato suggested that it would be difficult to improve flavonol accumulation via conventional breeding.

Nevertheless, the use of the primary gene pool has enabled the identification and use of wild species from the *Solanum* section *lycopersicum* as sources of variation. Following this approach, tomato flavonoid content has been increased using the wild tomato species *Solanum pennellii* Correl, in order to restore the flavonoid pathway in fruit flesh. With this objective, germplasm expressing chalcone isomerase in the flesh was selected and used in the development of hybrids with the cultivated species with higher levels of quercetin diglycoside [153].

Something similar happened with the improvement of anthocyanins in tomato. In fact, in the cultivated species anthocyanins are not produced in the fruit, but as a result of interspecific crossed in breeding programs, several mutants with anthocyanin accumulation in the fruit have been identified. The three mutants that can lead to increased anthocyanin content in the peel of the fruit are *Anthocyanin fruit* (*Aft*) *Aubergine* (*abg*) and *atroviolacea* (*atv*).

The *Anthocyanin fruit*, *Aft*, mutant was identified in crosses with *Solanum chilense* Dunal. This gene is located in chromosome 10 and its presence in tomato leads to increased levels of delphinidin, malvidin and petunidin, as well as higher levels of the flavonols quercetin, 3.6-fold, and kaempferol, 2.7-fold [37].

Sapir *et al.* [37] proved that *Aft* fruits not only showed high levels of anthocyanins in the skin and outer pericarp of the fruit, but also of the flavonols quercetin and kaempferol. They also showed that *Aft* is encoded by a single locus on chromosome 10 fully associated with *Anthocyacin1* (*Ant1*). *Slant1* was discovered in a T-DNA insertional mutagenesis program and was identified as a MYB transcription factor. Vegetative tissues of *Slant1* showed intense purple colour and fruits displayed purple spotting on the epidermis and pericarp. The overexpression of *Slant1* upregulated genes encoding enzymes of early and later steps of anthocyanidin biosynthesis as well as genes involved in the glycosylation and transport of anthocyanins into the vacuole [160]. It seems that the original allele from *S. chilense ScAnt1* would be more efficient in the production of anthocyanins than the tomato counterpart [161].

The *Ant1* paralog gene *SlAn2*, similar to Petunia *anthocyanin2*, is found in chromosome 10 and has also been related with the *Aft* mutant. *SlAn2* encodes an R2R3-MYB transcription factor and its overexpression in tomato results in increased anthocyanin accumulation in fruit peel [162]. Additionally, overexpressing fruits display ripening related phenotypes, with enhanced ethylene levels, reduced carotenoid accumulation and faster fruit softening. In fact, the authors found a concomitant accumulation of *Rin* (*Ripening inhibitor*) transcripts suggesting that the functions of *SlAn2* and *Rin* may be related. It has been recently studied that between *SlAnt1* and *SlAn2* only the latter acts as a positive regulator of anthocyanin synthesis in vegetative tissues under high light or low temperature conditions [163].

The *Aubergine* (*Abg*) mutant was identified in a cross with *Solanum lycopersicoides* [164]. This gene also relies in the chromosome 10 and it may be allelic to *Aft*. The difficulties in the management of *S. lycopersicoides* introgressions has hindered the development of allelic studies to confirm or discard this relation. In this sense, a paracentric inversion has been identified in the long arm of chromosome 10 of *S. lycopersicoides*, resulting in the absence of recombination events in this segment [165].

The recessive gene *atroviolacea* (*atv*), mapping in chromosome 7, was identified in a segregant population *S. cheesmaniae* (L. Riley) Fosberg [166]. It has a strongest effect on the accumulation of anthocyanins of vegetative tissues and has a limited effect on the fruit. The *atv* mutant shows an exaggerated response in the red broad band light, suggesting specificity for the phytochrome phyB1 high irradiance response pathway [167]. Thus, this mutation per se has no important effect on the accumulation of anthocyanins on the fruit. Nonetheless, it has been proved that in the double homozygous mutants *Aft Aft atv atv* a synergistic effect of both genes arises on the transcription of specific genes of the anthocyanin pathway, resulting in higher anthocyanin contents than in the individual mutants [168]. This effect also applies to the combination *Abg* with *atv*. In fact the best combinations to improve anthocyanin content in the fruit include *Abg atv atv* and *Aft Aft atv atv* in small fruits, with contents up to 415 and 116 mg 100g^-1^ fw respectively in the epidermis and subepidermis [36].

Another mutant, *anthocyanin free* (*af*), was identified in the fifties as a mutagenesis variant characterized by the lack of anthocyanin production in all plant tissues. Recently, Kang *et al.* [169] showed that *af* encodes SlCHI1, but complementation assays demonstrated that SlCHI1 not only complements flavonoid synthesis in the *af* mutant, but also complements a defect in terpenoid production, suggesting a link between both pathways.

None of these mutants result in the accumulation of anthocyanins in the fruit flesh. Alternatively, tomatoes with purple flesh have been obtained via genetic engineering. In this sense, the transcription *Del* and *Ros1* have been involved in the activation of the flavonoid pathway in tomato [147], as the expression of these factors from *Antirrhinum* resulted in the accumulation of purple anthocyanins in both peel and flesh. It seems that these factors would stimulate the transcription of the genes encoding PAL, CHI and F3’5’H. This upregulation of the flavonoid pathway and the opening of the anthocyanin gate (F3’5’H) resulted in anthocyanin levels up to 3 mg g^−1^ fw. Another possible success of the combination of both transcription factors may be related with the upregulation of genes involved in the side chain modification of anthocyanins and genes associated with the transport and accumulation in vacuoles [170]. Later works with the same genes obtained higher levels up 5.2 mg g^−1^ dry weight (dw), with petunidin-3-(*trans*-coumaroyl)-rutinoside-5-glucoside and delphinidin-3-(*trans*-coumaroyl)-rutinoside-5-glucoside representing an 86% of the total anthocyanins [171].

The enzyme F3’5’H would be in fact a key enzyme in restoring the accumulation of anthocyanins in tomato. In fact, the absence of anthocyanins in LC/C1 fruits was attributable primarily to an insufficient expression of F3’5’H, in combination with a strong preference of the tomato dihydroflavonol reductase (DFR) to use dihydromyricetin as a substrate [151].

### 4.3. Joint Accumulation of Carotenoids and Polyphenols

Following the objective of maximizing the functional value of tomato, several efforts have been made in order to maximize the accumulation of carotenoids and polyphenols in the same material. As stated in the carotenoids section, the use of the *high pigment* (*DDB1:hp-1, hp-1^w^, DET1: hp-2, hp-2^j^, hp-2^dg^*) mutants enables an improvement in the accumulation of both types of bioactive compounds. Lines carrying *hp-1* apart from increased carotenoid levels can show 13-fold higher levels of quercetin [172]. Long *et al.* [173] also found higher levels of chlorogenic acid in *hp-1*-lines (4.3-fold) and similar levels of hydroxycinnamic acids compared to a standard variety. The *hp-2^dg^* increased the level of quercetin aglycone 3.2-fold at the red stage, while the differences were not significant for naringenin chalcone [115]. It is not clear the connection of the two pathways, but considering that vitamin C contents are also higher in *hp-1* and *hp-2* plants [115,174] it could be possible that the expression of these alleles would trigger stress tolerance mechanisms activating the overproduction of antioxidants. Although both pathways seem to be completely independent, surprisingly, the combination of anthocyanin mutants with carotenoid defective mutants (*Beta, B*, and *yellow flesh*, *r*) results in lower total anthocyanin levels [36].

High pigment mutants have also been used in combination with anthocyanin synthesising mutants. Following this strategy, double homozygotes for *Aft* and *hp1* displayed a more than additive effect on the accumulation in anthocyanins and flavonols, with 5-, 19-, and 33-fold increase in the content of petunidin, malvidin and delphinidin respectively [37]. Mes *et al.* [36] also obtained higher anthocyanin levels combining *Aft* and *hp-1*, though the levels obtained were lower than the combination *Aft-atv*.

Stacking carotenoid and anthocyanin related genes is currently being used in the development of new cultivars with enhanced functional value. As an example, Sestari *et al.* [175] stacked the genes *hp-2*, *Aft* and *atv* into a commercial cultivar of cherry tomato. Cherry cultivars are selected due to their high ratio surface to volume, as carotenoids and polyphenols tend to accumulate in the external layers of the fruit, and more light is received by the peel increasing anthocyanin synthesis.

## 5. Conclusions

It is difficult to establish a clear link between a specific component of a complex diet with the prevention of different types of cancer. Especially, when their efficiency may depend on the matrix in which the bioactive compound is present. Consequently, supplementation studies may differ from their natural ingestion in food. Despite these difficulties, carotenoids and polyphenols have proved in different studies to play a role as functional compounds in the prevention of cancer. In this context, despite not outstanding for its nutritional value, the high level of consumption of tomato all year round makes it an important source of bioactive compounds. During the last decades, efforts have been placed in the breeding sector in order to develop new functional tomato varieties with increased levels of carotenoids and polyphenols. These materials would represent an alternative to isolated compounds found in dietary supplements, with the benefits of synergetic effects of these molecules with other phytochemicals presents in the natural matrix. The use of high pigment genes in combination with a restoration of anthocyanin accumulation in the fruit, especially in smaller materials such as cherry varieties, may represent a promising alternative to increment the intake of these bioactive compounds in their natural matrices in the near future. These new materials will obviously be an interesting tool to be combined with other recommendations in order to prevent or even to contribute to delay the progression of cancer.

## Figures and Tables

**Table 1:** Typical composition (mg 100 g^−1^ fresh weight) in tomato ripe fruits of carotenoids and polyphenols, (adapted from [24,25,26,27]).

Carotenoid	Concentration	Polyphenol	Concentration
Lycopene	7.8–18.1	Naringenin chalcone	0.9–18.2
Phytoene	1.0–2.9	Rutin	0.5–4.5
Phytofluene	0.2–1.6	Quercetin	0.7–4.4
β-Carotene	0.1–1.2	Chlorogenic acid	1.4–3.3
γ-Carotene	0.05–0.3	Caffeic Acid	0.1–1.3
δ-Carotene	0–0.2	Naringenin	0–1.3
Lutein	0.09	Kaempferol-3-rutinoside	0–0.8
Neurosporene	0–0.03	*p*-Coumaric acid	0–0.6
α-Carotene	0–0.002	Ferulic acid	0.2–0.5
Neoxanthin	-	Kaempferol	0–0.2
Violaxanthin	-	Myricetin	-
Anteraxanthin	-	Cyanidin	-
Zeaxanthin	-	Pelargonidin	-
		Delphinidin	-

**Table 2:** Mutants used in tomato breeding related to carotenoid accumulation.

Altered Activity	Mutant/Gene	Fruit Colour	Details
Single step of biosynthesis pathway	*r* (*yellow flesh*)	Yellow fruits	Null mutation of PSY-1 gene arrests carotenoid synthesis and the yellow colour is due to rutin accumulation
	*t* (*tangerine*)	Orange fruits	Defective CRTISO enzyme. Prolycopene (orange colour) is accumulated
	*og* (*old gold*) and *og^c^ (old gold crimson*)	Intense red fruits	Frameshift mutations originating defective CYC-B Lycopene is accumulated at the expense of β-carotene
	*B* (*Beta*)	Orange fruits	Mutation in the promoter of CYC-B gene (increased transcription). β -carotene increases at the expense of lycopene
	*Del* (*Delta*)	Orange fruits	LCY-E transcription is increased, and more δ-carotene is produced at the expense of lycopene
	*hp-3* (*high pigment 3*)	Intense red fruits	Defective ZE mutant. Biosynthesis of ABA is decreased, resulting in increased plastid division and higher accumulation of carotenoids
Regulation	*hp-1, hp-1^w^* (*high pigment 1*)	Intense red fruits	Mutation of DD1 homolog. Altered light regulation. Increased total carotenoids, vitamin C and polyphenols
	*hp-2, hp-2^j^, hp-2^dg^* (*high pigment 2*)	Intense red fruits	Mutation of TDET1. Altered light signal-transduction machinery. Increased total carotenoids, vitamin C and quercetin
	*Ip* (*Intense pigment*)	Intense red fruits	Promotion of phytochrome signal amplification. Increased soluble solids and total carotenoids

**Table 3:** Mutants used in tomato breeding related to polyphenol accumulation.

Altered activity	Mutant/gene	Fruit colour	Details
Regulation	*Y* (*yellow*)	Pink fruits	Mutation of SlMYB12, transcription factor involved in the regulation of phenylpropanoid/flavonoid pathway. Lack of accumulation of naringenin chalcone in fruit peel
	*Aft* (*Anthocyanin fruit*)	Purple fruits (external)	Probable regulation of flavonoid/anthocyanidin synthesis in fruits. Increased levels of flavonols, delphinidin, malvidin and petunidin
	*Abg* (*Aubergine*)	Purple fruits (external)	Possible allele of *Aft*.
	*atv* (*atroviolacea*)	Light purple fruits (external)	Possible role in the phytochrome *phyB1* high irradiance response pathway. Strong accumulation of anthocyanins in vegetative parts. Small effect on fruit, but it has synergic effects with *Aft*
	*hp-1, hp-1^w^* (*high pigment 1*)	Intense red fruits	Mutation of DD1 homolog. Altered light regulation. Increased total carotenoids, vitamin C and polyphenols
	*hp-2, hp-2^j^, hp-2^dg^* (*high pigment 2*)	Intense red fruits	Mutation of TDET1. Altered light signal-transduction machinery. Increased total carotenoids, vitamin C and quercetin
